# Regeneration of β cells from cell phenotype conversion among the pancreatic endocrine cells

**DOI:** 10.1002/cdt3.15

**Published:** 2022-03-02

**Authors:** Tianjiao Wei, Rui Wei, Tianpei Hong

**Affiliations:** ^1^ Department of Endocrinology and Metabolism Peking University Third Hospital Beijing 100191 China

**Keywords:** cell trans‐differentiation, endocrine pancreas, β cell dedifferentiation, β cell regeneration

The prevalence of diabetes has increased dramatically, with over 537 million adults affected worldwide.^1^ In both type 1 and type 2 diabetes, pancreatic islet β cell dysfunction is common pathogenesis and substantial decrease in β cell mass can induce clinical manifestations.^2^ Therefore, replenishing β cells, which includes transplantation of exogenous β cells and promotion of β cell regeneration *in situ*, is an important therapeutic strategy. Pancreatic cells, especially the endocrine cells, share similar developmental paths, gene expression profiles, and epigenetic landscapes.^3^ The endocrine pancreas has five types of islet cells; β cells, α cells, δ cells, PP (pancreatic polypeptide) cells, and ε cells (producing insulin, glucagon, somatostatin, pancreatic polypeptide, and ghrelin, respectively), and each type of cell functions and interacts with others accordingly.^4^ It has been shown that pancreatic endocrine cells can convert into each other.^5^ Therefore, cell phenotype conversion between pancreatic endocrine cells is a promising strategy for the recovery of β cell mass.

## 1 RE‐DIFFERENTIATION OF THE DEDIFFERENTIATED PANCREATIC β CELLS

Following physiological or pathological stress, β cells lose their mature, healthy characteristics and revert to progenitor‐like cells, which is called cell dedifferentiation.^6^ Accilli and his colleagues first revealed the β cell dedifferentiation phenomenon in diabetic mice, and found that the transcriptional factor FoxO1 participates in the process.^6^ Subsequently, scientists extended this concept to humans by using histological and single‐cell transcriptomic analyses.^7^, ^8^ Treatments of diabetes with diet control,^9^ gastric bypass surgery,^10^ and several antidiabetic therapies including intensive insulin therapy,^11^ sodium‐glucose co‐transporter type 2 (SGLT2) inhibitor,^12^ and glucagon‐like peptide‐1 (GLP‐1)‐based therapies^13^ have been reported to promote β cell re‐differentiation in type 2 diabetic rodents and in cultured primary islets. Apart from the anti‐hyperglycemic strategies, anti‐inflammatory agents and inhibition of oxidative stress can also induce β cell identity restoration. For instance, salsalate and resveratrol prevent β cell dedifferentiation in rodents and rhesus monkeys *in vivo*, and in primary human islets *in vitro*.^14^ Moreover, mesenchymal stem cells and the stem cell‐derived particles (including exosomes or cytokines) can reverse β cell dedifferentiation.^15^, ^16^ However, continued intervention might be required to maintain the mature phenotype and avoid the progressive loss of β cells.

## 2 TRANSDIFFERENTIATION OF PANCREATIC α CELLS

Pancreatic α cells are the second most abundant cells in islets (about 18% in mice and 38% in humans); the α cell mass is preserved or even increased in diabetes,^17^ which makes α cells an attractive option for β cell regeneration. Bi‐hormonal cells co‐expressing glucagon and insulin are observed in patients with impaired glucose tolerance, type 2 diabetes, and type 1 diabetes,^18^ suggesting that pancreatic endocrine cells have plasticity and α cells own the potential to transform into β cells. By using α cell lineage‐tracing technology, Thorel et al.^19^ have proved that α cells can spontaneously convert into β cells under conditions of almost complete ablation of β cells. Furthermore, gene manipulation, including forced expression of β cell‐specific genes (e.g., *Pax4*,^20^ or *Nkx6.1*,^21^ or *Pdx1* and *MafA* ^22^), or ablation of α cell master genes (e.g., *Arx* ^23^) in α cells triggers the trans‐differentiation of α cells to β cells during fetal or postnatal development in mice. Human islets from nondiabetic or type 2 diabetic donors transduced with *PDX1* and *MAFA* also show conversion of α cells to β cells and a glucose‐lowering effect in the diabetic recipient mice after the transplantation.^24^ Interestingly, several antidiabetic therapies have the ability to promote α‐to‐β cell conversion. Many research groups have demonstrated that glucagon receptor blockage, a promising novel antidiabetic strategy, increases β cell mass through α‐to‐β cell conversion in type 1 diabetic mice.^25^, ^26^ The α cell‐derived β cells account for approximately 15% of the insulin‐producing cells in type 1 diabetic mice, and the glucose‐lowering effect can be maintained for at least 3 weeks after withdrawal of the intervention,^26^ which provides new insight into the clinical development of this strategy for treating diabetes. Moreover, we also show that dapagliflozin, an SGLT2 inhibitor, promotes α‐to‐β cell conversion in type 2 diabetic mice, although this conversion only accounts for a small fraction of the regenerated β cells.^12^ Enhancement of GLP‐1 signaling by several approaches, including transfection of recombinant adenovirus expressing GLP‐1, GLP‐1 receptor agonists or dipeptidyl peptidase 4 (DPP‐4) inhibitors (which prevent degradation of GLP‐1 by DPP‐4), can induce α‐to‐β cell conversion in type 1 and type 2 diabetic rodents,^13^, ^27^, ^28^ and increase β cell‐like gene expression in a subcluster of human α cells.^13^ Either glucagon receptor blockage or dapagliflozin can upregulate GLP‐1 levels in the circulation and islets, which is suggestive of the importance of GLP‐1 in mediating α‐to‐β cell conversion.^12^, ^25^, ^29^ Nevertheless, more research is needed to reveal how α‐to‐β cell conversion is initiated, which factors are involved in the process, and how to promote the process.

Notably, defective α cells (particularly in terms of their glucose‐sensing abilities) in diabetes might cause the functional deficits of trans‐differentiated β cells. The previous studies mostly used toxins (including streptozotocin, alloxan, and diphtheria toxin) to induce β cell loss, and transcriptomic profiles of α cells in these conditions did not seem to be different from normoglycemic control mice.^30^ In these models, the newly formed α cell‐derived β cells are able to improve glycemic control and have similar expression profiles of key genes (including *Ins1*, *Ins2*, *Pdx1*, *Mafa*, *Ucn3*, and *Nkx6.1*) to the normal β cells, suggesting that the trans‐differentiated β cells are at least partly functional.^31^ On the contrary, in the naturally developed animal models of diabetes, α cell mass and function differ from those in the normal control animals.^17^ Nevertheless, in these diabetic models, rare α cells could trans‐differentiate into β cells. Besides, research on gene expression profiles and function of trans‐differentiated β cells is rare and usually superficial owing to the low numbers. It remains unclear whether the function of the regenerated β cells is normal. Lineage‐tracing technology together with single‐cell transcriptomic analysis might help us determine this.

## 3 TRANSDIFFERENTIATION OF PANCREATIC δ CELLS

Pancreatic δ cells, which mainly secrete somatostatin, are located in the islet mantle similar to the α cells. Some somatostatin and insulin co‐expressing cells can be found in normal individuals and patients with type 2 diabetes,^32^ which suggests the possibility of cell conversion between δ cells and β cells. In juvenile mice with β cell ablation, δ cells (instead of α cells) can dedifferentiate, proliferate, and finally differentiate to functional β cells.^33^ The δ cell‐derived β cells account for 90% of the insulin‐producing cells, and lead to recovery from diabetes rapidly,^33^ suggesting a huge potential for β cell regeneration. The δ‐to‐β cell conversion during pre‐puberty offers a new perspective for therapy of children with type 1 diabetes. However, the trans‐differentiation of δ cells to β cells is hard to realize in adults; the number of δ‐to‐β cell conversion in adult type 1 diabetic mice is lower, constituting 17% of insulin‐producing cells, compared with that in juvenile type 1 diabetic mice (about 90%).^33^

Recently, Gribben et al.^34^ observed that Ngn3‐positive progenitors located in the pancreatic ducts express somatostatin, and then convert into β cells during homeostasis in adult mice. A FoxO1 inhibitor increases the number of the δ cell‐derived β cells, accounting for 93% of the insulin‐producing cells after β cell ablation in adult mice.^33^ Our group demonstrates that glucagon receptor blockage increases δ cell mass and induces δ‐to‐β cell conversion in adult type 1 diabetic mice.^35^ Nevertheless, bi‐hormonal cells co‐expressing somatostatin and insulin might also be derived from the progenitors rather than the intermediate cells through direct conversion between mature δ cells and β cells. Overexpression or activation of Pax4 might be a trigger factor,^36^ but the potential mechanism is still unclear.

## 4 TRANSDIFFERENTIATION OF PANCREATIC PP CELLS AND ε CELLS

Pancreatic PP cells and β cells are thought to be derived from a common precursor.^37^ In PP cell lineage‐tracing mice with massive β cell loss, PP cells can convert into β cells, constituting about 40% of the insulin‐producing cells.^37^ In addition, human PP cells with ectopic expression of *PDX1* and *MAFA* can be reprogrammed to insulin‐producing cells *in vitro*.^24^ To date, there are no reports on transdifferentiation of ε cells to β cells. Interestingly, pancreatic ε cells are found to express Ngn3, a marker of endocrine progenitor,^38^ implying the potential of cell reprogramming. However, the small fraction (2%–3% of pancreatic endocrine cells) and lack of detailed knowledge of the functions of both PP cells or ε cells makes the PP‐to‐β or ε‐to‐β cell conversion more difficult to study and less practical for clinical translation.

## 5 CONCLUDING REMARKS AND FUTURE PERSPECTIVES

Collectively, pancreatic β cells can regenerate from cell phenotype conversion among the pancreatic endocrine cells. As for the conversion path, re‐differentiation of the dedifferentiated β cells takes the spotlight because of its accessibility to mature β cells. Transdifferentiation of α cells to β cells attracts lots of attention because of larger cell mass and compensatory hyperplasia in α cells under diabetic conditions.^17^ In contrast, studies on the phenotype conversion between δ cells and β cells or between PP cells and β cells barely scratch the surface. As for the conversion method, gene manipulation often results in the mature β cell phenotype, but has safety concerns in clinical translation. The clinically available drugs or potential new agents are easy to administer and translate into clinical application. Although the regenerated insulin‐producing cells can secrete insulin and regulate blood glucose levels following the above pharmacological interventions, they may only gain part of the characteristics of bona fide β cells.^39^ Therefore, it is highly needed to reveal potential mechanisms and discover new targets. In addition, combination treatment may be more effective and efficient for β cell regeneration but needs to be verified.

There are several concerns that need to be addressed. First, different lineage‐tracing models may be associated with varying levels of accuracy and efficiency of the transgenic animals, thereby leading to variable results.^40^ Second, there are molecular, structural, and functional differences as well as developmental disparities between rodent and human islets.^41^ Although several antidiabetic drugs and small molecules possess the ability to reverse β cell dedifferentiation and/or promote the transdifferentiation of other pancreatic endocrine cells to β cells in rodents, their effects in humans seem to be low.^42^ More efforts should be made in human β cell regeneration, including 3D culture and organoid. Third, isolated human islets differ from the islets *in vivo*.^43^ However, we cannot evaluate islet cell phenotype and mass in humans owing to the lack of reliable tracing technology. Fourth, we must pay attention to the function of the original cells. For example, hypoglycemia should be avoided when α cells are used as the starting cells. Fifth, protecting the regenerated β cells from immune system attack is vital for type 1 diabetes. Although the neogenic β cells show hypoimmunogenic characteristics with a long survival in autoimmune diabetic mice,^26^, ^31^ combination of immunomodulators (e.g., anti‐CD3 antibodies^44^) with regenerative agents may be a promising strategy to increase β cell mass for a longer period in treating type 1 diabetes but it needs supportive evidence. Lastly, pancreatic cell transdifferentiation as a strategy to restore β cell mass is very important, but not well developed often with the low transdifferentiation efficiency and the immature transdifferentiated phenotype. The technical difficulties are probably the biggest challenges and uncertainties regarding future expectations for clinical application. Despite all these concerns, the β cells derived from other pancreatic endocrine cells can realize β cell regeneration in situ, which may fulfill the coordinated function of islet cells when compared with transplantation or production of β cells in other sites. The current and ongoing studies are on the right track, and more efforts are needed in the same direction.

## CONFLICT OF INTERESTS

The authors declare no conflict of interest. Professor Rui Wei and Tianpei Hong are members of Chronic Diseases and Translational Medicine editorial board and are not involved in the peer review process of this article.
